# Evaluation of Encapsulated Liver Cell Spheroids in a Fluidised-Bed Bioartificial Liver for Treatment of Ischaemic Acute Liver Failure in Pigs in a Translational Setting

**DOI:** 10.1371/journal.pone.0082312

**Published:** 2013-12-18

**Authors:** Clare Selden, Catherine Wendy Spearman, Delawir Kahn, Malcolm Miller, Anthony Figaji, Eloy Erro, James Bundy, Isobel Massie, Sherri-Ann Chalmers, Hiram Arendse, Aude Gautier, Peter Sharratt, Barry Fuller, Humphrey Hodgson

**Affiliations:** 1 University College London Institute for Liver & Digestive Health, University College London Medical School, Royal Free Hospital Campus, Hampstead, London, United Kingdom; 2 Division of Hepatology, Department of Medicine, University of Cape Town, Groote Schuur Hospital, Cape Town, Western Cape, South Africa; 3 Department of Surgery, University of Cape Town, Groote Schuur Hospital, Cape Town, Western Cape, South Africa; 4 Department of Anaesthetics, University of Cape Town, Groote Schuur Hospital, Cape Town, Western Cape, South Africa; 5 Department Neurosurgery, Red Cross Children's Hospital, University of Cape Town, Cape Town, Western Cape, South Africa; 6 Biochemistry Department, University of Cambridge, Cambridge, United Kingdom; 7 Division of Surgery and Interventional Science, University College London Medical School, Royal Free Hospital Campus, Hampstead, London, United Kingdom; Institute for Frontier Medical Sciences, Kyoto University, Japan

## Abstract

Liver failure is an increasing problem. Donor-organ shortage results in patients dying before receiving a transplant. Since the liver can regenerate, alternative therapies providing temporary liver-support are sought. A bioartificial-liver would temporarily substitute function in liver failure buying time for liver regeneration/organ-procurement. Our aim: to develop a prototype bioartificial-liver-machine (BAL) comprising a human liver-derived cell-line, cultured to phenotypic competence and deliverable in a clinical setting to sites distant from its preparation. The objective of this study was to determine whether its use would improve functional parameters of liver failure in pigs with acute liver failure, to provide proof-of-principle. HepG2cells encapsulated in alginate-beads, proliferated in a fluidised-bed-bioreactor providing a biomass of 4–6×10^10^cells, were transported from preparation-laboratory to point-of-use operating theatre (6000miles) under perfluorodecalin at ambient temperature. Irreversible ischaemic liver failure was induced in anaesthetised pigs, after portal-systemic-shunt, by hepatic-artery-ligation. Biochemical parameters, intracranial pressure, and functional-clotting were measured in animals connected in an extracorporeal bioartificial-liver circuit. Efficacy was demonstrated comparing outcomes between animals connected to a circuit containing alginate-encapsulated cells (Cell-bead BAL), and those connected to circuit containing alginate capsules without cells (Empty-bead BAL). Cells of the biomass met regulatory standards for sterility and provenance. All animals developed progressive liver-failure after ischaemia induction. Efficacy of BAL was demonstrated since animals connected to a functional biomass (+ cells) had significantly smaller rises in intracranial pressure, lower ammonia levels, more bilirubin conjugation, improved acidosis and clotting restoration compared to animals connected to the circuit without cells. In the +cell group, human proteins accumulated in pigs' plasma. Delivery of biomass using a short-term cold-chain enabled transport and use without loss of function over 3days. Thus, a fluidised-bed bioreactor containing alginate-encapsulated HepG2cell-spheroids improved important parameters of acute liver failure in pigs. The system can readily be up-scaled and transported to point-of-use justifying development at clinical scale.

## Introduction

Both acute and acute-on-chronic liver failure are well recognised. Liver failure resulting from viral hepatitis, obesity, alcohol abuse, and drug-induced injury presents major clinical problems. Whilst organ transplantation is potentially curative, there is a huge gap between organ availability and supply. Since the liver has the ability to repair and regenerate given time, alternative therapies are sought.

Liver support devices, bioartificial livers, aim to provide temporary synthetic and detoxificatory function buying time either for liver repair and regeneration, or acting as a bridge to transplantation [1]. Purely artificial liver support devices, for example, those based on albumin dialysis did not significantly improve survival in recent clinical trials [2], [3], likely reflecting the complex functional repertoire of the liver that only a device containing a biological component can provide.

To achieve adequate function in a bioartificial liver (BAL), the choice of liver cells, the format of biomass provision, and bioreactor design are paramount.

Several bioartificial liver machines have been trialled utilising primary porcine or human hepatocytes at a scale equivalent to 5–10% in vivo human liver mass [4]. We and others have argued that a successful bioartificial liver to treat patients should be of human origin, for reasons including safety, zoonosis risk and potential species incompatibility in protein/protein receptor interaction. Thus whilst pig cells are readily available they have disadvantages in all these respects including protein-protein compatibility [5]. Primary human hepatocytes are not readily available, as good-quality explant livers are used for transplantation. An alternative is the use of human-derived liver cell lines, such as the ELAD system using the C3A subclone of HepG2 cells and other experimental devices with Hepa RG cells [6] and a fetal liver cell line [7].

We have developed a BAL biomass comprising HepG2 cells in alginate beads of ∼500–600 um diameter. In this system, single HepG2 cells in the cell-beads rapidly multiply to form multicellular spheroids of aggregated cells, with close cell-to-cell contact mimicking that in vivo. In this system we showed that, in contrast to conventional monolayer culture in which various functions are only expressed at low levels, alginate encapsulation imposing three-dimensional growth increases a broad range functions up to ten-fold, often approximating in vivo levels [8]. The cytochrome P450 3A function in our 3-dimensional culture format is within the range of freshly isolated primary human hepatocytes in culture, although the urea cycle is incomplete [9], [10]. Moreover, in this culture format, HepG2 cells create a rich extracellular matrix, and this combines with the three dimensional format to maximise function [11].

A key concern in BAL design is the presence or absence of a barrier to diffusion between cells and patient plasma, as BAL designs should maximise transfer of toxic metabolites from the blood to the cells for detoxification, and molecules synthesised by the biomass (eg clotting factors, carrier proteins) must readily reach the patient circulation.

To house alginate-encapsulated HepG2 cells, we and others developed a fluidised bed-based bioreactor (FBB) [12]–[15]. In this, cells are kept in continued motion under the force of upward flow of media and downward force of gravity, and this differs from packed bed reactors in which cells are immobile. Thus the fluidised bed bioreactor increases mass transfer. Moreover, the FBB imposes no physical filtration barrier between the biomass and the circulation, the plasma directly accessing the alginate hydrogel beads, and can be readily up-scaled for clinical usage by simply increasing the volume of alginate beads and geometry of the biomass chamber

For this study, we charged the device with alginate beads containing 4–6×10^10^ HepG2 cells, equivalent to 20–40% of human hepatocyte biomass (based on the assumption of 1–2×10^11^ hepatocytes in 1.2–1.5 kg human liver) to treat pigs. This desired biomass was estimated from data suggesting survival is possible with 10–30% residual liver mass, but taking into account that the milieu of acute liver failure plasma may be detrimental to biomass viability [16].

The objective of this study was to assess its value in a large animal model. We used pigs with acute ischaemic liver failure surgically induced (20–30 kg bw; liver weights of 900–1300 g). Importantly, the experiment compared animals treated with the cell-containing BAL to animals undergoing an identical surgical and anaesthetic protocol with the use of an acellular BAL i.e. an identical bioreactor containing alginate beads from which HepG2 cells were absent.

The alginate beads were generated at a central expert tissue culture facility and transported to a distant hepatology centre for assessment in ALF, and to enable this, a short-term cold-chain system was developed.

Using this model, we demonstrated improvements in several key clinical parameters of acute liver failure, including intracranial pressure (ICP), blood clotting function, bilirubin conjugation, acidosis and protein synthesis, not found when animals were treated with the BAL without cells.

## Methods

### Ethics Statement

Animal experiments were performed under approval from University of Cape Town (UCT), Faculty of Health Sciences Animal-Ethics Committee, according to the UCT Animal Unit health/welfare guidelines. Application No.:009/019, 26/03/09.

### Production of alginate-encapsulated hepatocytes and culture to liver cell spheroids for BAL investigation

The method for production of the cellular component of the BAL has been described elsewhere [12]. In brief, it can be divided into two steps (a&b).

### (a) HepG2 Monolayer Cell Culture for ELS production

#### HepG2 verification

Cells were assessed independently for cell line identity using two methods, microsatellite genotyping and DNA bar coding (HPA, Porton Down UK); for mycoplasma using qPCR and two cell culture-based assays, Hoechst dye with indicator Vero cells, and agar plate growth of conditioned media from cultured cells, according to Eu Pharm Current Edition Section 2.6.7 Mycoplasmas (Vitrology, Scotland UK); and in house for several human pathogens. Sterility was assessed via direct inoculation methods according to Eu Pharm Current Edition; Section 2.6.1Sterility and USP Current Edition <71>Sterility tests (Vitrology, Scotland, UK).

#### Monolayer culture conditions

HepG2 cells were obtained from the ECACC (Wiltshire, UK) and maintained in modified MEM-alpha medium (Gibco, Paisley, UK) supplemented with 10% FBS (Hyclone, Loughborough, UK), 100 IU/ml penicillin and 0.1 mg/ml streptomycin. Medium was changed every 2–3 days. Cells were counted under light microscopy using a haemocytometer; viability was assessed using trypan blue dye exclusion.

### (b) Alginate Encapsulation and Culture of ELS

Alginate encapsulation of HepG2 cells was performed as described previously. In brief, 80% confluent monolayer cultures were trypsinised and encapsulated in 1.0% alginate (alginic acid sodium salt *Macrocystis pyrifera* kelp) [17]. Alginate beads, containing cells were resuspended in culture medium at a ratio of 1∶58. Culture expansion in a fluidised bed bioreactor was carried out for 11 days [12]. Medium was changed every 2–3 days unless otherwise stated. Bead diameters and cell numbers were assessed on day 0 and at time of harvest.

### Short term cold chain

Alginate beads containing HepG2 cell-spheroids were transported from the laboratory where they were cultured to the point of use between distinct layers of oxygenated perfluorodecalin (PFC) and culture medium in a ratio of 1∶1∶10 at ambient temperature, with 50% air volume. Antioxidants (500 IU/ml catalase (Sigma-C9322), 0.85 mM Trolox (Sigma-238813) and 3 mM N-acetyl cysteine (Sigma-A8199), valine (Sigma-V0500), 25mMHEPES buffer (Gibco-15630) were added to reduce oxidative stress, and maintain pH ∼7.4. Prior to use, beads were washed with normal saline containing 1.7 mM CaCl_2_ and re-suspended in heparinised pig plasma. Viability was determined before and after PFC storage using dual staining with fluorescein diacetate (FDA, live cells) and propidium iodide (PI, dead cells), quantified using image-analysis.

### BAL circuit set-up

The BAL chamber contained 1100 ml alginate bead suspension comprising 4.29±1.6×10^10^ (±SD, n = 6) HepG2 cells as optimally functional spheroids. Heparinised normal pig plasma was used to prime both primary and secondary circuits. The biomass was fluidised at 400–600 ml/min to achieve a 2-fold bed-height. Temperature was maintained at 37°C; oxygenation was provided in the chamber via porous tubing at 1000 cc/min.

### Surgical Procedures

Large 25–30 kg White-x-Landrace female pigs [n = 13] were acclimatized in the Animal Unit 7 days prior to surgery. Prior to inducing ischaemic acute liver failure, pigs were fasted overnight. They then received continuous anaesthesia throughout with induction using doses of zoletil, butorphanol and medetomidine for tracheal intubation, followed by maintenance with isoflurane, oxygen and nitrous oxide via an endotracheal tube. Fluid management comprised 0.9%saline at 20 ml/kg/hr and boluses of 0.9%saline to maintain stroke volume variation <15% and a CVP of 10 mmHg. Glucose was monitored hourly, and 5% or 10% glucose in normal saline was given if glucose levels fell below 4 mmol/L to maintain normoglycaemia at a glucose level of ≥4 mmol/L. No extra blood products were given during the process. Intracranial pressure and brain oxygenation monitoring catheters were inserted via a burr hole, and in some animals intra-cerebral microdialysis catheters. A 2-hour period was allowed to reach a steady-state baseline. Continuous vasopressor support was not given, however at the time of connection of pig to BAL a small bolus dose of Ornipressin (<2units) was titrated to counteract the transient drop in blood pressure observed on connection. Animals remained under continuous anaesthesia until the end of the procedure and were humanely euthanased with 3 g potassium chloride as required by the Animal Ethics Committee.

### Ischaemic acute liver failure

**Figure 1:**
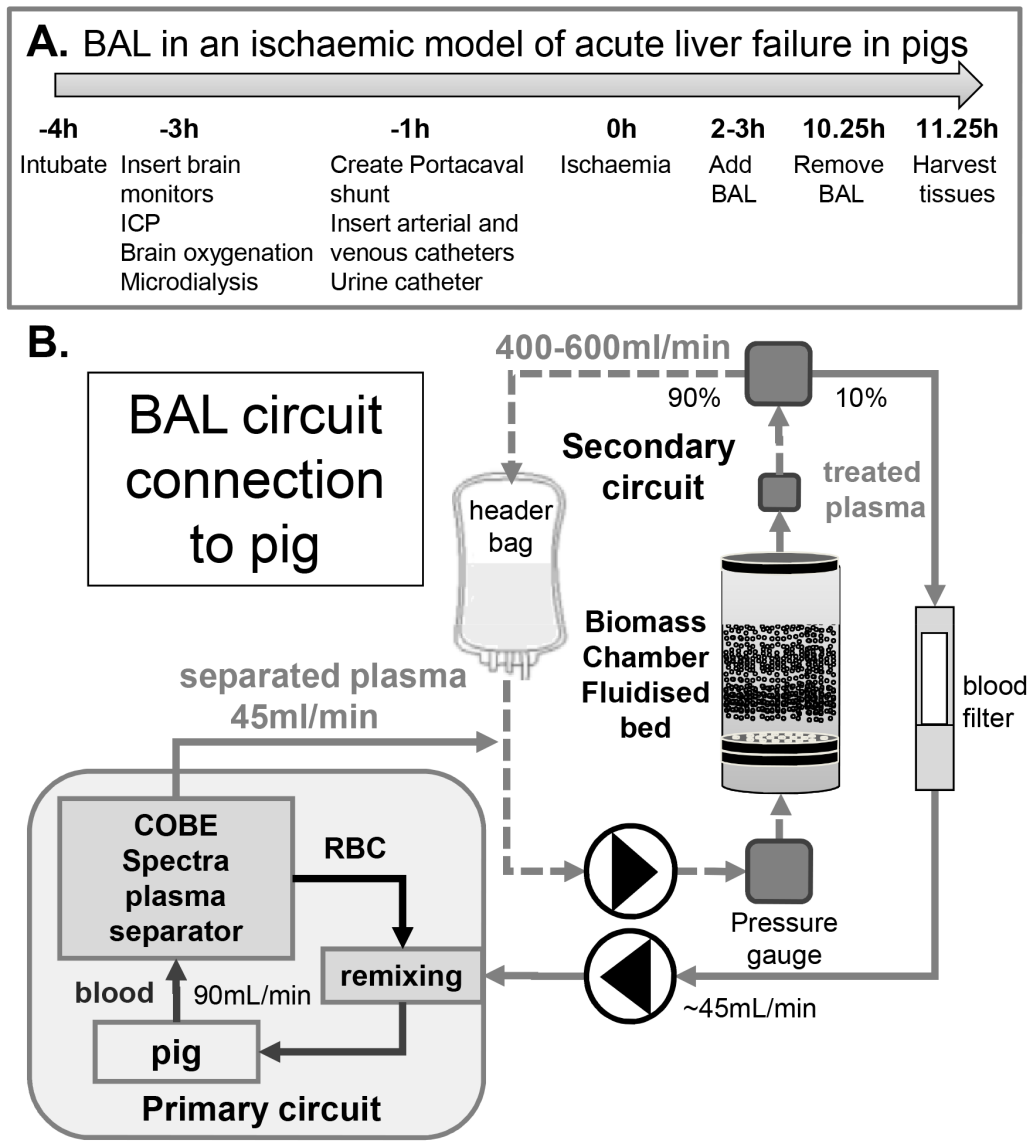
Protocol of ischaemic acute liver failure model. (**A**) Pigs were treated with the bioartificial liver machine 2–3 h after establishing ischaemic damage. After anaesthesia, brain monitoring catheters were inserted, prior to establishment of porta-caval shunt and arterial, venous and urine catheter placement. (**B**) **Schematic of the bioartificial liver machine:** The fluidised-bed bioreactor chamber containing either cell bead biomass (Group 1) or empty beads for control (Group2), was attached to the pig via a plasmapheresis machine. Blood was obtained from the pig via the splenic vein at 90 ml/min and separated from cellular components with a Cobe Spectra Plasma separator, providing plasma at a flow rate of ∼45 ml/min in a primary circuit, feeding into the BAL secondary circuit at ∼400–600 ml/min. Whole blood was returned to the pig via the plasma-separator at 90 ml/min, combining the “treated ”plasma with the cellular component.

In the supine position, pressure monitoring vascular catheters were inserted into the femoral artery and the internal jugular vein. The abdomen was opened via a midline incision and ligamentous attachments of the liver divided. The hepatic artery and bile duct were ligated and divided, portal vein and infrahepatic vena cava dissected. A side-to-side portacaval shunt was created and liver rendered totally ischaemic by ligating the portal vein above the shunt [18], [19]. Catheters were inserted into the splenic (venous outflow) and external jugular vein (venous return) for attachment to the extracorporeal circuit of the BAL via a COBE-Spectra plasmapheresis machine (Figure 1). The pigs were connected to the COBE-Spectra with 90 ml/min blood flow (40–47 ml/min plasma) in a primary circuit, ∼2.5 hours after establishment of ischaemia. Separated plasma entered the secondary circuit at 400–600 ml/min and was then recirculated several times through the BAL chamber; there was continuous return of plasma to the patient via the COBE-Spectra blood circuit at 90 ml blood/min (Figure 1). The BAL remained connected to the pig for up to 8 hours, prior to rinseback/disconnection, and animals were killed and tissues harvested for histology. Two groups of pigs were compared, each with ischaemic acute liver failure: Group 1 (Gp 1) were treated with a BAL containing cell-bead biomass, Group 2 (Gp 2, non-functional BAL) treated identically, but with empty-alginate-bead completely devoid of cell bio-mass. Death was defined by a mean arterial pressure of <40 mmHg for 30 minutes, if this occurred before the end of the 8 h bioartificial liver procedure.

### Monitoring

Haemodyamic monitoring consisted of routine ECG, pulse oximetry and end tidal carbon dioxide. Continuous cardiac output (PCCI) was monitored using the PiCCO-plus (Pulse Contour Continuous Cardiac Output, Pulsion Medical Systems SE, Germany) via CVP measurement in right internal jugular vein and femoral artery cannulae. Biochemical parameters were measured by an accredited Chemical Pathology laboratory (Pathcare, South Africa) as would occur in a clinical patient setting. Blood samples were taken into heparinase cups for thromboelastography (TEG) coagulation parameter assessment (TEG, Haemonetics, MA, USA). Codman Express Intracranial pressure (ICP-Codman-Raynham-MA, USA) probes and Lycox brain oxygenation probes (Integra, NJ, USA) monitored brain function. Blood Glucose was estimated hourly, and blood gases at multiple time points during the procedure.

Human proteins were measured in pig plasma using ELISA for human albumin, alpha-1-antitrypsin, alpha-1-acid glycoprotein, fibrinogen and prothrombin. These antibodies showed no cross reactivity with porcine proteins. Amino acids were analysed by high-performance-ion-exchange chromatography [20].

## Results

### Cell line characterisation

The HepG2 cell bank used, was proven to be identical with the original deposited cell source, was entirely of human origin, was not contaminated by any mycoplasma species and met sterility criteria required for human use in a cell therapeutic. Moreover, after a screen, more extensive than used for clinical liver transplantation, the following viruses were undetected: Parvovirus, HHV6, HSV/VZV, HSV 1&2, EBV, adenovirus, the full respiratory panel (Flu A&B, RSV, Rhino, Paraflu 1–3, hMPV), Hepatitis B, Hepatitis C, and Polyoma viruses JC and BK.

### Viability of biomass

The viability of encapsulated cell mass was 90.70±2.3% at the end of the 11 day conditioning culture. We have previously shown that exposure to mild cold-chain storage as described in Methods did not alter the viability of the BAL biomass for up to 48 h [12]. Therefore, in the current trial on ALF, an upper limit of 48 h cold chain was set. After transport conditions, the BAL viabilities were 88.56±8.53%, which was not significantly different from the starting viability (93.25±8.26%, n = 6,mean ±SD, NS). Moreover, proliferation continued so that the overall viable cell number remained the same (at harvest, 4.29±1.6×10^10^; after 2 days on PFC 4.54±1.49×10^10^ cells.

### Surgical model for ALF

Liver and body weights were the same for both studied groups: Group 1 (Gp1 cell bead BAL) 26.8±2.3 kg bw, n = 6, mean±SD; liver weight 1173±122 g vs. Group 2 control (Gp2 empty bead non-functional BAL) 26.7±3.4 kg bw, n = 7, mean±SD; liver weight 1113±209 g). Fig 1A shows the time course for the establishment of the ischaemic ALF model; Fig 1B illustrates the BAL circuit, and its integration into conventional plasmapheresis technology.

### Chemical pathology in response to ischaemic ALF

**Figure 2:**
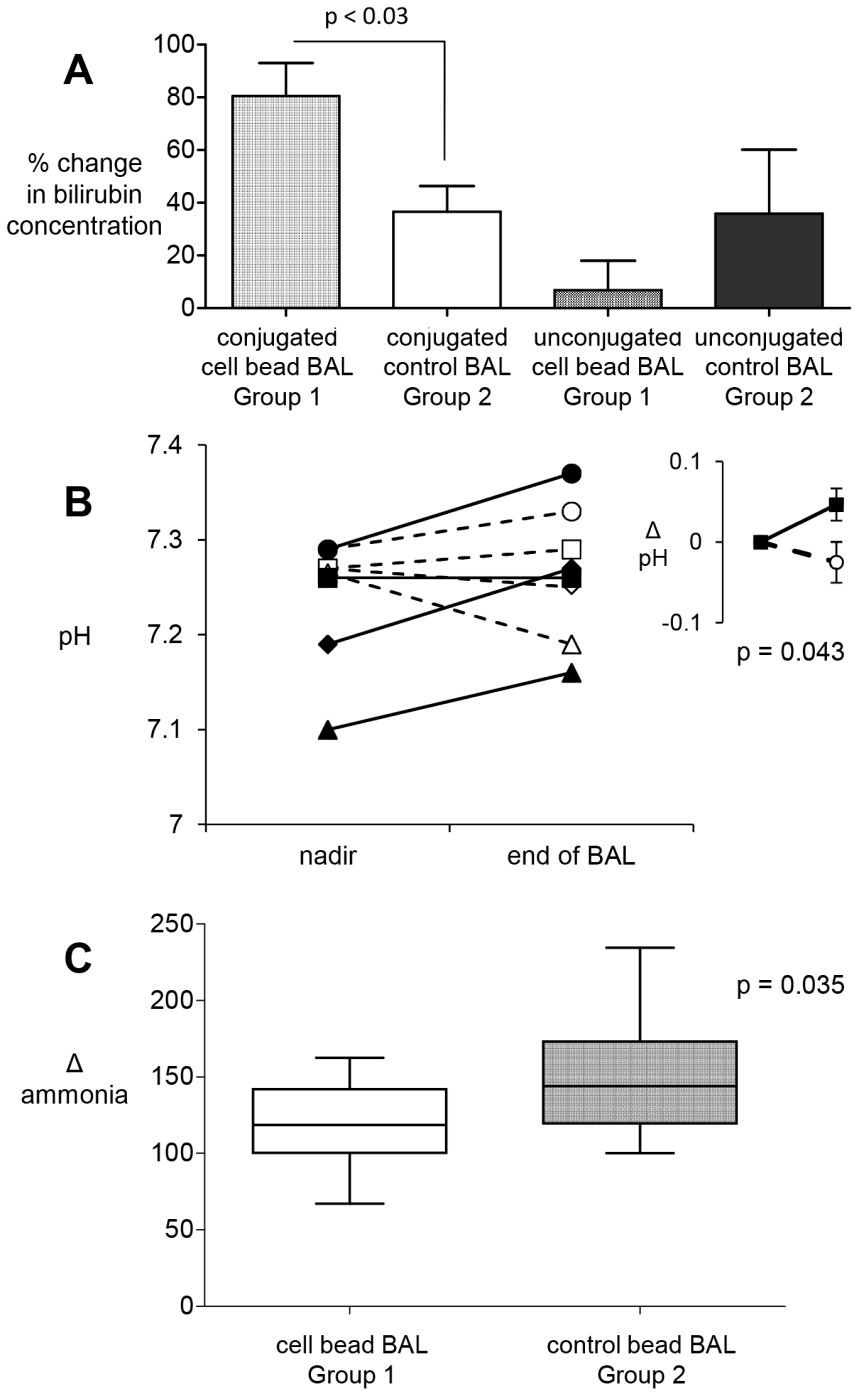
Efficacy of HepG2-Fluidised-bed bioreactor treatment in pigs with ischaemic acute liver failure: biochemical response. **A) Bilirubin conjugation in pigs with ischaemic acute liver failure.** Conjugated bilirubin concentrations (µmol/L) increased and unconjugated bilirubin concentrations decreased in Group 1 pigs, when attached to the cell-bead BAL; in contrast there was neither increase in conjugated bilirubin nor decrease in unconjugated bilirubin in Group 2 control animals, Group 1 n = 6; group-2 n = 5; p-value, unpaired 2-tailed t-test, mean±SEM. **B). Change in acidosis in pigs with ischaemic acute liver failure, after BAL treatment.** pH values dropped after ischaemic insult to a nadir in both groups. Blood pH was assessed in a blood gas analyser. Pigs in the Cell-bead BAL (Group 1 solid line) showed an increased pH towards normal at the end of BAL treatment whereas pigs treated with the control empty bead BAL (Group 2 -----) showed little improvement; inset shows group averages (n = 4, mean±SEM unpaired, one-tailed t-test). **C) Blood ammonia levels in pigs 4 h after BAL treatment.** values show a change in ammonia from 0.5 h after ischaemia (normalised to 100%) during BAL treatment. Concomitant with a decrease in ICP in Group 1, ammonia levels decreased (cell BAL) cf. group 2 (Control empty bead BAL); Group 1 n = 6, mean 120±8; Group 2 n = 7, mean 150±11. Statistics: two-tailed unpaired t-test, p = 0.035.

Table 1 illustrates the liver function in both ALF groups after ischaemia prior to BAL treatment. The data represent the range of measurements routinely performed in chemical pathology to assess patients presenting clinically with ALF. At the start of BAL therapy, the groups were comparable in the measured indices (Table 1). With the establishment of total liver ischaemia via the portocaval shunt and ligation of the hepatic artery: ammonia increased at the time the portocaval shunt was created; international normalised ratio (INR), lactate and bilirubin increased after ∼2 hours of ischaemic ALF. After addition of BAL in Group 1 (active BAL +cell biomass), conjugated bilirubin increased with a concomitant decrease in unconjugated bilrubin; in contrast, in Group 2 (non-functional - empty beads without cells BAL), unconjugated bilirubin increased progressively, with little increase in conjugated bilrubin (Figure 2A). Parameters of acid-base balance acidosis (pH, acid-base-excess, lactate, bicarbonate) showed the expected changes associated with ischaemic hepatic damage in both groups; improvements in these indices of circulatory physiology were seen in Group 1, whereas these parameters did not improve in Group2 (non-functional BAL). Improved pH control from nadir values early in ALF to end of BAL treatment reached statistical significance for Group 1 (p = 0.03, n = 4, unpaired, equal variances, 1-tailed) but as expected, this was not achieved in Group 2 (p = 0.4), see Figure 2B. Ammonia levels were lower in Group 1 but not in Group 2 (Fig 2C).

**Table 1:** Biochemistry, liver function tests and haematology in pigs with ischaemic acute liver failure.

	Group 1 (Cell-bead BAL) average time after ischaemia = 2.55 h							Group 2 (Control empty bead BAL) average time after ischaemia = 2.33 h							
**TEST**	Pigs:	1	2	3	4	5	6	7	8	9	10	11	12	13	*normal range*
ALBUMIN-S	g/L	**10**	**10**	**10**	**11**	**9**	**8**	10	11	10	9	9	9	10	*37*–*52*
ALK. PHOSPHATASE	µ/L 37′C	**137**	**129**	**115**	**224**	**97**	**124**	155	164	123	103	161	153	63	*40*–*120*
ALT (SGPT)	µ/L 37′C	**30**	**28**	**40**	**45**	**43**	**29**	50	34	34	41	55	30	15	*10*–*32*
AMMONIA PLASMA	µmol/L	**532**	**632**	**424**	**267**	**425**	**276**	395	559	430	271	643	299	293	*11.0*–*32.0*
ANION GAP	mmol/l	**19**	**23**	**17**	**15**	**18**	**16**	19	16	22	15	21	17	12	*5*–*15*
AST (SGOT)	µ/L 37′C	**77**	**117**	**96**	**172**	**513**	**129**	65	56	55	42	405	130	14	*10*–*32*
BILI CONJ.-S	µmol/L	**2**	**3**	**2**	**2**	**3**	**2**	2	3	2	3	2	2	2	*0*–*8*
BILI UNCONJ.-S	µmol/L	**7**	**7**	**7**	**6**	**8**	**5**	5	4	8	6	8	7	5	*2*–*14*
BILIRUBIN TOTAL-S	µmol/L	**9**	**10**	**9**	**8**	**11**	**7**	7	7	10	9	10	9	7	*2*–*20*
CALCIUM-S	mmol/L		**2.04**	**2.05**			**2.03**	2.34	2.45		2.3	2.11	2.2	1.46	*2.10*–*2.60*
CO2-S	mmol/L	**20.6**	**12.9**	**16.4**	**21.1**	**15.4**	**16.8**	19.4	24.6	18.2	16.3	11.9	18.9	12.6	*22.0*–*30.0*
CREATININE-S	umol/L	**111**	**111**	**82**	**86**	**95**	**80**	73	91	136	76	115	118	64	*39*–*91*
FIBRINOGEN	g/L	**0.5**	**0.4**	**0.8**	**0.5**	**0.8**	**0.7**	0.5	0.3	0.5	0.7	0.8	0.9	0.6	*2.2*–*5.0*
GAMMA GT-S	µ/L 37′C	**33**	**34**	**35**	**37**	**25**	**27**	36	25	37	19	47	26	16	*5*–*40*
GLUCOSE	mmol/L		**6.2**	**3.9**	**2**	**4.9**	**2**	5.4			7.4	8.4	19.9	7.2	
HAEMATOCRIT	l/L	**0.39**	**0.34**	**0.4**	**0.36**	**0.32**	**0.29**	0.4	0.35	0.38	0.39	0.25	0.31	0.21	*0.35*–*0.45*
HAEMOGLOBIN	g/dL	**12.7**	**10.9**	**13.2**	**11.8**	**10.5**	**9.2**	13.4	11.6	12	12.9	7.9	10.6	6.7	*11.5*–*15.5*
INR		**2.8**	**2**	**3.4**	**2.6**	**2.6**	**3.8**	2	3.2	4.2	2.4	1.5	3	3.9	*0.9*–*1.3*
LACTATE PLASMA	mmol/L	**5.9**	**10.1**	**4.8**	**4.5**	**9.6**	**3.3**	3	5.8	8.5	4.9	11.1	5.2	4.2	
PLATELET COUNT	x 10∧9/l	**154**	**192**	**271**	**335**	**237**	**178**	224	236	323	238	211	260	190	*140*–*420*
PROTHROMBIN TIME	sec	**26.6**	**19.5**	**32.1**	**25.1**	**24.6**	**35.9**	19.4	30.2	39.9	22.5	14.9	28.2	36.9	*9.8*
SODIUM-S	mmol/L	**139**	**142**	**139**	**142**	**144**	**142**	140	145	141	136	146	140	147	*136*–*144*
TOTAL PROTEIN-S	g/L	**40**	**41**	**41**	**45**	**35**	**29**	42	45	41	40	40	41	26	*60*–*82*
UREA-S	mmol/L	**3.1**	**4**	**3.3**	**1.1**	**3.3**	**1.5**	1.6	2.4	2.1	1.8	3.2	3	1.5	*2.5*–*6.7*
WHITE CELL COUNT	x 10∧9/l	**26.6**	**13.9**	**13.3**	**20.7**	**22.5**	**10.3**	17.8	18.6	17.5	12.1	17.3	13.9	9	*4.0*–*11.0*

Group 1  =  animals to be treated with a Cell-Bead BAL (bold) and Group 2  =  animals to be treated with a control empty bead non functional BAL (plain text). Data are from blood samples taken at the start of the BAL addition, representing baseline acute liver failure (normal range in italics).

### Cerebral physiology in response to ALF

**Figure 3:**
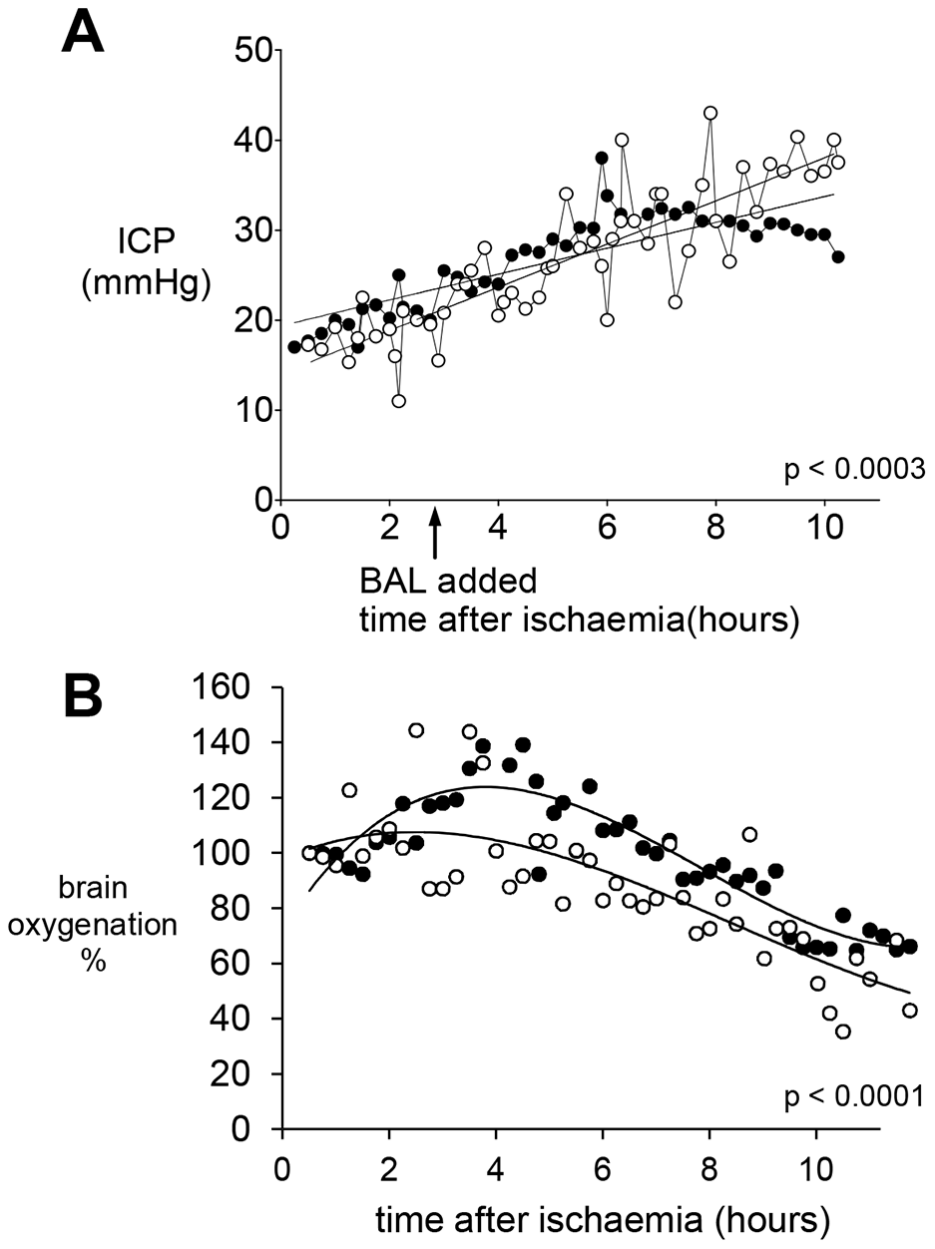
Brain parameters in pigs with ischaemic acute liver failure. **A**) Intracranial Pressure (ICP) using a Codman catheter was recorded every 15 min, in mmHg. The rise after established acute ischaemic liver failure was halted when pigs were attached to the cell bead BAL (Group 1, n = 5, closed circles ·), whilst it continued to increase in Group 2 (empty bead BAL open circles o), n = 7. **B**) Brain oxygenation, normalised to 100% at time of ischaemia. There was an increase in brain oxygenation in Group 1 cell bead treated pigs (solid circles ·) compared with Group 2 (open circles o). Statistics compared the slopes of the line between groups with 95% confidence limits.

**Figure 4:**
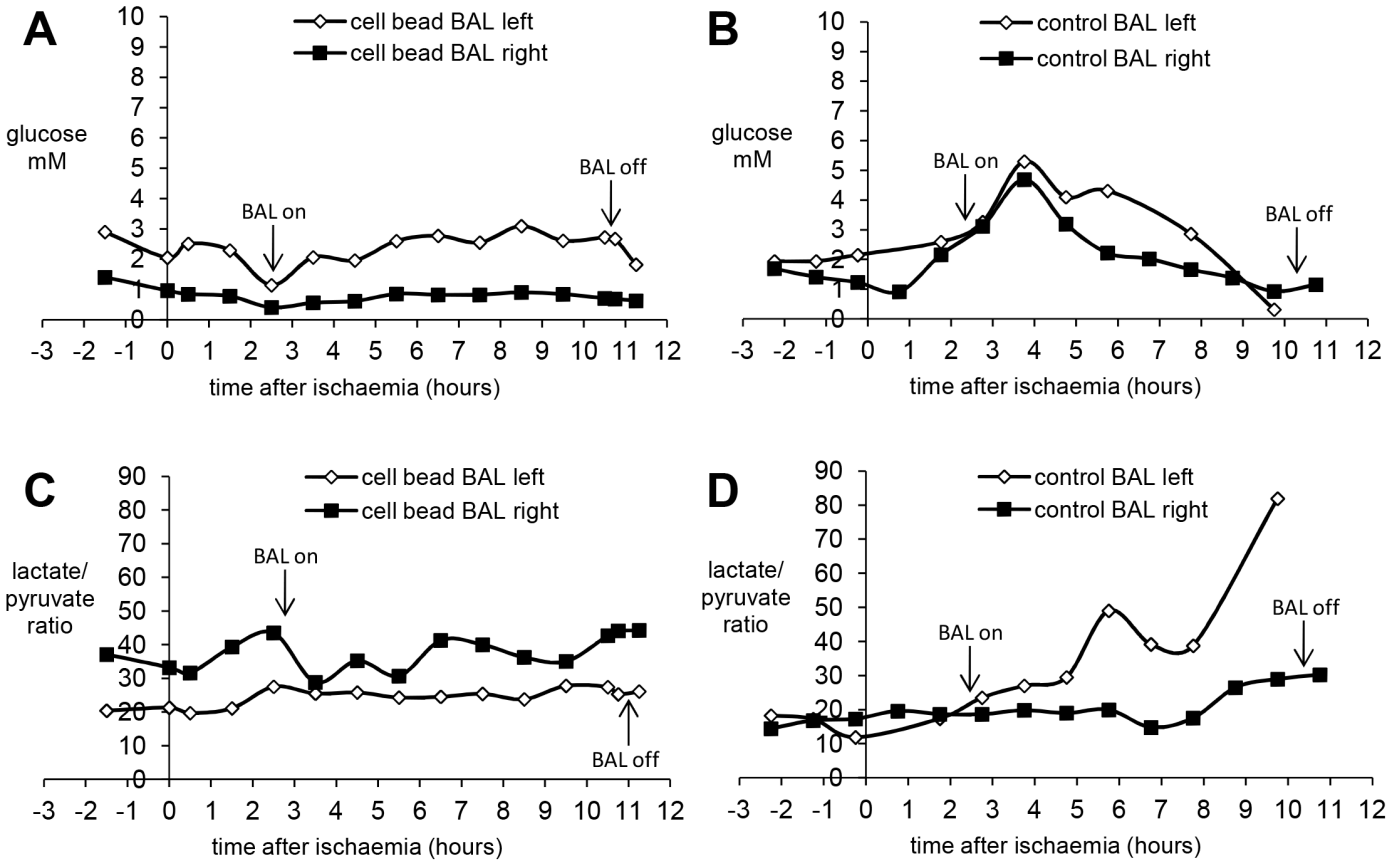
Parameters of brain homeostasis during BAL treatment of pigs with ischaemic liver failure: Brain metabolites. Microdialysate samples (18 µl) were collected from catheters on each side of the brain. Fig 4 **A&B** show glucose levels in the brain during the course of treatment using a cell-bead BAL (**4A**) or an empty bead BAL control (**4B**). Left and right refer to left and right brain hemispheres. 30 minute microdialysates were collected; measurements made hourly. Fig **4C&D** show the lactate:pyruvate ratio indicating ischaemic damage in cell-bead BAL (**4C**) and empty bead BAL control (**4D**); note that 4D shows different results on each side of the brain, perhaps indicating patchy brain ischaemia. These results are representative of the 4 pigs in which microdialysis was performed, two in Group 1 and two in Group 2.

In both groups, intracranial pressure (ICP) increased after induction of ischaemic liver damage. BAL addition initiated a brief immediate decrease in ICP in both groups, suspected to be associated with dilution of the pig circulating plasma volume with normal plasma from the primed system in the COBE. Thereafter, ICP continued to rise in the control BAL group (Group 2), but not in the cell-bead BAL treated group (Group 1) (Fig 3A). The brain oxygenation response was more variable. After ischaemia was induced, there was a progressive decrease in brain oxygenation in some animals, whilst others exhibited a period of presumed hyperemia before decline. (Fig 3B). After BAL addition, there was an improvement in oxygenation in Group 1 vs. Group 2. Analysis of brain metabolites was achieved for four pigs. Fig.4 shows a typical trace for glucose, and lactate:pyruvate ratios in each side of the brain demonstrating a decrease in glucose and increase in lactate:pyruvate ratios in the control empty BAL treated pig compared with glucose maintenance and no increase in lactate:pyruvate ratio in cell bead treated pig.

### Plasma amino acids

Derangement in plasma amino acid balances are a feature of ALF. 10 amino acids increased from start to end of treatment in Group 1 and 13 amino acids increased in Group 2; 4 amino acids decreased in Group 2, whilst in Group 1, 7 amino acids decreased (Table-2). The change from start to end of treatment was significant in each group.

**Table 2:** Amino acid concentrations in pig plasma before and after treatment.

amino acid	cell-bead BAL start	cell-bead BAL end	control empty bead BAL start	control empty bead BAL end	amino acid	cell BAL	control BAL
Asp	18.5±1	18.3±2	15.7±2	24.0±4	**Asp**	**decrease**	**increase**
Thr	214±32	287.6±30	148±35	267±57**	Thr	increase	increase
Ser+Asn+Gln	541±50	974±52**	509±35	989±139*	Ser+Asn+Gln	increase	increase
Glu	203±54	178±31	227±33	232±56	**Glu**	**decrease**	**increase**
Gly	1188±109	1583±77*	1221±65	1729±182*	Gly	increase	increase
Ala	796±104	1139±164*	856±111	1156±157**	Ala	increase	increase
(Cys)2	30.3±6	27.4±7	24.7±2	34.9±6	**(Cys)2**	**decrease**	**increase**
Val	362±35	223±15*	312±41	253±22	Val	decrease	decrease
Met	56.6±5	111±13*	47.9±4	102±18*	Met	increase	increase
Ile	168±21	80±10*	140±21	100±13*	Ile	decrease	decrease
Leu	268±26	155±17*	237±33	187±20*	Leu	decrease	decrease
Tyr	104±12	169±7**	77.5±11	154±22**	Tyr	increase	increase
Phe	106.3±6	195±21*	87.3±12	198±31**	Phe	increase	increase
His	109.5±7	131±10	98.4±10	137±16**	His	increase	increase
Lys	437±28	557±67	300±35	523±89*	Lys	increase	increase
Arg	115±28	0**	139±11	8.8±6**	Arg	decrease	decrease
Pro+Cys	312±33	499±36**	280±24	483±60**	Pro+Cys	increase	increase

Amino acids, measured in pig plasma by ion exchange HPLC were quantified in Group 1 and Group 2 animals. Results expressed as µmol/L, mean±SEM. Differences between start and end of treatment for each group (Group 1 cell-bead BAL n = 6, and Group 2 empty bead non-functional control BAL n = 5), are show with statistical differences: *p<0.05; **p<0.02, t-test, paired, 2-tailed. Three amino acids showed differences in response to treatment with the BAL comparing Group 1 and Group 2.

### Synthetic function

**Figure 5:**
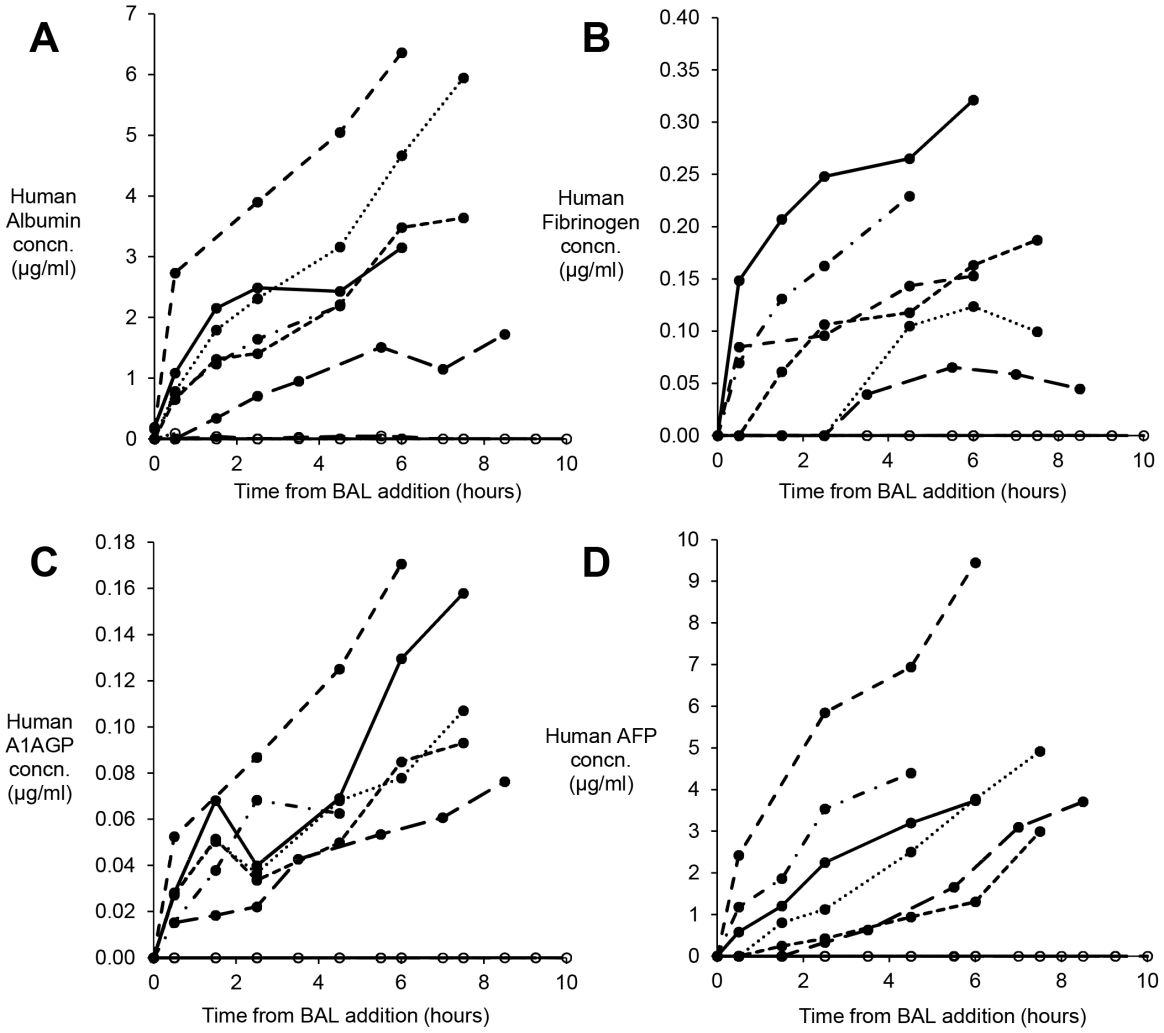
Human proteins in pig plasma after BAL treatment. Pig blood samples were assessed by ELISA for specific human protein content during treatment; Group 1 cell bead treated BAL (solid symbols), compared with Group 2 empty bead control treated BAL (open circles) (**A**) Albumin (**B**) Fibrinogen, (**C**) Alpha-1-acid glycoprotein (A1AGP) and (**D**) Alphafoetoprotein (AFP). Results expressed as µg/ml pig plasma: mean±SEM n = 6. No human protein was detected in Group 2 animals, establishing the specificity of the assays for human proteins.

Human proteins were detected in plasma from all Group 1 pigs, and none in Group 2 (Figure 5). Albumin, alpha-1-acid glycoprotein fibrinogen and alphafoetoprotein (AFP) were detectable at all time points after BAL addition. Plasma human Alpha-1-antitrypsin and prothrombin levels, were assessed only at 4 h after BAL, and in Group 1 were 0.557±0.05 µg/ml and 0.291±0.04 µg/ml respectively; Group 2 control pigs exhibited no human proteins.

### Clotting

**Figure 6:**
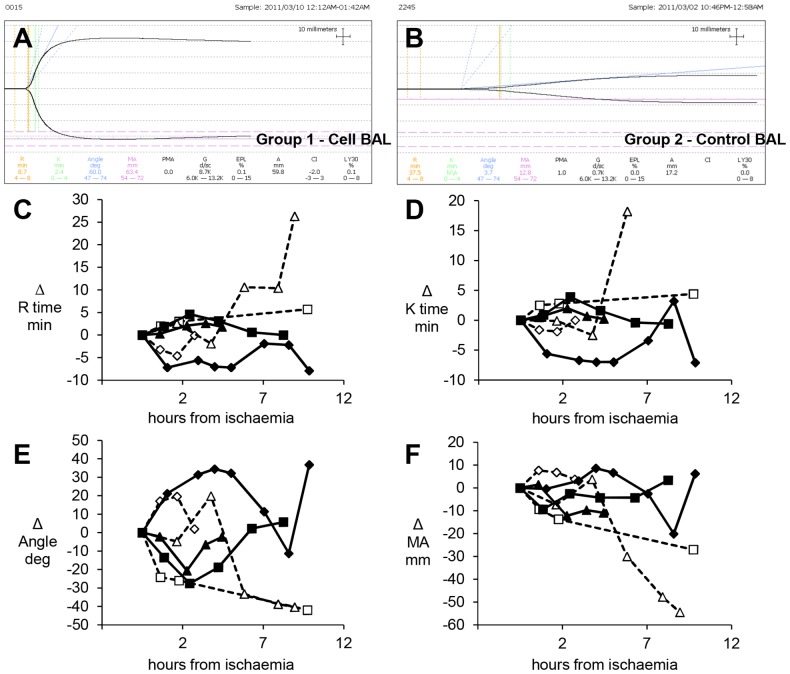
Thromboelastography (TEG) measurements of coagulation. **A&B) Example of TEG traces**: in cell-bead treated BAL Group 1 **(A)** and empty bead control treated BAL Group 2 **(B). Clotting parameters quantified (C-F):** Thromboelastography of blood during treatment with either Cell bead treated BAL (solid symbols) or empty bead control treated BAL (open symbols); n = 3: results for each pig are shown as a change from the start of ischaemia (normalized and denoted as 0 on Y axis), demonstrate changes in R-time **(C)**, K-time **(D)**, angle **(E)** and maximum amplitude (MA) **(F)**. BAL was added ~2.5h later. The four parameters describe the overall clotting reaction. The R-time (min) reflecting fibrin formation rate, is functionally dependent on clotting factors, notably fibrinogen; reported as the reaction time from placement in the cup to 2mm amplitude on the tracing, thus, the higher the number the longer it takes for formation of clot. In both groups initially the time taken for fibrin formation is increased during liver failure; as the BAL effects an improvement in Group 1, the time taken for fibrin to form is reduced; in contrast in Group 2 the time continues to increase **(C)**. The K time, reflecting viscoelasticity, is the time taken from the R time to the point where the trace amplitude reaches 20mm, indicating intrinsic clotting factor activity, as well as fibrinogen and platelet function. Similar to the R time, the longer the time the less intrinsic clotting factor activity; pigs in Group 1 show a decrease in K time with continued treatment whereas those in Group 2 continue to increase **(D)**. The alpha angle (angle of slope of r to k denotes rate of clot formation); in Group 1, pigs had an increased angle with BAL treatment, in Group 2 animals the angle decreased with time **(E)**. MA is the maximum amplitude on the trace, reflecting the absolute strength of the clot; Group 1 pigs showed a stronger clot compared with Group 2 animals **(F)**.

Thromboelastography (TEG) measurements were used to assess clotting dysfunction and possible correction by the active BAL biomass. Not all pigs developed severe coagulopathy, however, in those that did cell-bead Group 1 animals demonstrated a restoration of clotting as estimated by TEG K and R times, angle and MA values, whilst Group 2 showed no improvement (Figure 6). In both groups initially coagulation parameters worsened during liver failure. BAL treatment from 2.5 h effected a general improvement in Group 1, in contrast to Group 2 where parameters continued to decline. For example, the time taken for fibrin formation is increased during liver failure; as the BAL caused an improvement in Group 1, the time taken for fibrin to form was reduced; in contrast in Group 2 the time continued to increase. The K time, indicating intrinsic clotting factor activity, as well as fibrinogen and platelet function decreased in Group 1 with continued treatment whereas those in Group 2 continued to increase. The alpha angle in Group 1 pigs increased with BAL treatment, in Group 2 animals the angle decreased with time. The maximum amplitude on the trace (MA), indicated the absolute strength of the clot was stronger in Group 1 compared with Group 2 animals (see also Fig 6A&B).

### Survival

As intended, all animals died whilst under anaesthesia and observation, without recovery being attempted. Average survival time after establishment of ischaemic ALF in Group 1 (active BAL +cells biomass) was 10.46+/−0.7 h, that of Group 2 (control non-functional BAL) was 8.63+/−1.4 hours. This was a non-significant trend towards longer stabilisation in Group 1. Extraneous supportive interventions from the anaesthetists (as might normally be administered alongside clinical bioartificial liver suport therapy) were precluded by the study design.

### Haemodynamic variables

As expected central blood pressure dropped significantly on creation of the porto-systemic shunt as part of the ALF model, and on initial connection to the COBE, but this rapidly corrected. Cardiac output increased predictably as liver failure progressed, largely due to a progressive drop in systemic vascular resistance (SVRI), which was similar in both groups. Haemodynamic data, which was not significantly different between the two groups, is summarised in Fig S1. Extra-vascular lung water (EVLW) also increased indicating acute lung injury in the Group 2, but less so in Group 1 (Fig-S2A-) during the first 4 hours of BAL treatment, albeit not statistically significantly different. Cerebral perfusion pressure (CPP) did not differ between the two groups (Fig S2B).

Histological analysis after death demonstrated patchy ischaemic necrosis throughout the liver, without engorgement of gut, similar in both groups. Patchy brain ischaemia was also demonstrable. Histology of the other organs was normal. (Fig S3).

## Discussion

Liver transplantation is the established treatment for acute liver failure, and is effective by providing complete functional liver support almost immediately. However, future trends, spurred on by the lack of donor organs, justify the exploration of alternatives, such as cell based therapies. We have investigated the role of a liver cell line cultured in a 3-dimensional format housed in a fluidised bed bioreactor to provide temporaray liver support in a near clinical model.

Important goals in liver support therapy include provision of synthetic and detoxification functions, restoration of blood coagulability, protection from intracranial hypertension as well as a positive impact on the inflammatory response of acute liver failure, with an indication that regeneration of the injured liver is occurring [1]. To assess all these in a single animal model is challenging, since ideally both a predicatable rate of onset of liver damage, and the potential for survival should occur.

The model of surgical ischaemic acute liver failure in pigs mimics well the clinical scenario where there is a universal increase in plasma ammonia, acidosis and raised intracranial pressure in contrast to some unpredictability in drug-induced acute liver failure models in out-bred pigs [21]. With the surgical model all animals will suffer ischaemic acute liver damage, although clearly the predictable deterioration and irreversible liver damage does not provide an appropriate model to investigate any impact on survival. The clinical outcome of this ischaemic model is a consequence both of deficient hepatic function due to the necrotic liver combined with deleterious effects of substances released from the necrotic liver into the circulation. The release of substances from the liver is evidenced by the rising plasma transaminase levels demonstrating leakage from the liver into the plasma. The deleterious consequences of the presence of a necrotic liver on patients' systemic physiology has been clearly indicated by demonstration of clinical improvement when the necrotic liver is removed in Acute Liver failure prior to salvage liver grafting. (Prolonged anhepatic state after early liver graft removal.[22] We have demonstrated improvement in important aspects of acute liver failure in pigs treated with our active BAL biomass. Acidosis, conjugation of bilirubin, and ammonia improved. The decreased ammonia levels were indeed unexpected since HepG2 cells are known to lack a full urea cycle, (being deficient in Ornithine transcarbamylase at the mRNA level) as do C3A cells [9], [10]. One explanation for the decreased ammonia may be the consumption of some amino acids at a significant rate by the biomass, removing the substrate for ammonia generation. The amino acid profiles did differ between the two groups after treatment. Notably also, HepG2 cells have a second amino acid transport systems (System ASC – sodium dependent amino acid transporter) not found in primary hepatocytes that results in rapid glutamine uptake, which may also enhance ammonia elimination [23], [24].

Synthetic function was clearly demonstrated by the active BAL biomass, with human proteins assayed appearing at progressively rising levels in the pigs' plasma in Group 1. The lack of any increases in Group 2 confirms the specificity of the assays. It is noteworthy, that in future clinical application production of AFP by the biomass may provide a helpful specific tracer as this is rarely present in plasma of patients with acute liver failure. Fibrinogen and prothrombin are likely contributing to the improvement of clotting function, which we measured by Thromboelastography (TEG), assessed to be a better measure of clotting dysfunction than prothrombin time and aPTT in a model of hypothermia and haemorrhagic shock in swine [25]. TEG measures several parameters of clotting function, reflecting viscoelastic properties of blood, platelet activation, fibrin formation and retraction of the clot [26], [27]. The R-time reflects fibrin formation rate, functionally dependent on clotting factors, and particularly fibrinogen; for their derivation see Fig 4. The K time, reflects viscoelasticity, intrinsic clotting factor activity, as well as fibrinogen and platelet function. The alpha angle and MA reflect the absolute strength of the clot. In the animals in whom clotting was grossly deranged, those in the Group 2-control showed no correction, whilst in Group 1 this returned to near normal values. This improvement was noted after several hours of BAL treatment consistent with the appearance of human fibrinogen in pig plasma [25].

The provision of freshly synthesised albumin by the biomass may be of particular significance. Much of previous artificial, non-biological, liver machine work is based on a form of albumin dialysis. Whilst none so far has improved survival, there is symptomatic relief, for example, of pruritis in some patients, and that is associated with the removal of certain toxins by the albumin. However, one issue that has emerged is the source and thus biological efficacy of the albumin in such systems and its fitness-for-purpose to act as a carrier protein to aid detoxification. Commercially isolated/prepared albumin has an altered affinity for toxins which makes it considerably less effective. In contrast the HepG2 cells are continuously making endogenous nascent albumin at the time of need, and in our system the per cell production is indeed equivalent to that calculated from human in vivo albumin production data [8]; this nascent albumin has been unaffected by the preservatives e.g. caprylate, used in preparing commercial albumin and which has been shown to adversely affect toxin binding [28].

A striking clinical observation was that the intracranial pressure increase, resulting from ischaemic acute liver damage, was arrested in pigs treated with the cell-bead BAL. Cerebral perfusion pressures were identical in both groups. This resolution of ICP in Group 1 could have had a trivial explanation had it been associated with a parallel change in cerebral perfusion pressure, since diminished brain perfusion would also ultimately decrease ICP. However, this was not the case; both groups had identical profiles for cerebral perfusion pressure, indicating a specific decrease in intracranial pressure. Interestingly, in the clinical situation an ICP value of ≥30 mmHg in man is associated with brain herniation and a poor prognostic outcome; the values in the control group rose above 35 mmHg whereas those in the cell treated group plateaued lower than 30 mmHg.

Clearly the improvements in biochemical and systemic manifestations of acute liver failure reported here are only partial. Obviously, in a pig model, human derived cells do not substitute ideal function. However, despite possible incompatibilities, we demonstrated important improvements in parameters relevant to acute liver failure using Hep G2 cells.

Whilst human primary hepatocytes might be considered the ideal cells for use in man, and have been used [29], accessing such cells on a rapid, sufficient and regular basis is unlikely. Primary hepatocytes rapidly lose differentiated function in culture and scarcely proliferate. Proliferating hepatocytes derived from stem cells are a potentially attractive source, but are not yet a reality. Current possibilities therefore, for a human source, are cell-lines, derived either spontaneously, e.g. HepG2 (or C3A), Hepa RG, or by immortalization, generally viral transformation. Whilst HepG2 cell lines do not express the full *in vivo* repertoire of function when cultured as conventional monolayers [9], their function is markedly up-regulated when the same cells are cultured in a three-dimensional format mimicking in-vivo architecture [8], [30]. Many 3-D culture approaches necessitate use of products of animal origin e.g. collagen, matrigel etc.; one advantage of our 3-D culture system is use of the non-animal product alginate, derived from seaweed, providing the substrate for cell attachment required by epithelial cells. Notably this system differs from the use of polycation-coated alginate, in which after encapsulation and polycation-coating, the hydrogel is dissolved; furthermore that process introduces a diffusion barrier, e.g. Poly-L-lysine molecular weight cut-off is 70,000Da. [31]–[33].

It is also worth noting that several other cell bioreactors introduce a molecular barrier. In the ELAD and other systems using hollow-fibre technology to house the biomass, cells are separated from patient blood or plasma by a semi-permeable membrane [34]–[37], which, however, limits exchange and mass transfer. One notable exception is the AMC BAL, in which plasma contacts cells directly [38], [39].

Previously, we demonstrated that [17], [40] HepG2 cells in alginate perform synthetic and detoxification functions in the presence of both normal and acute liver failure plasma, and in a small animal model of acute liver failure induced with acetaminophen [41], improved haemodynamics using a small-scale packed-bed bioreactor. Here, using the superior technology of a fluidised-bed bioreactor on a near human scale (∼5×10^10^ cells in 1100 ml), we have demonstrated improvements in a pig acute liver failure model and thus provided proof-of-principle for alginate-encapsulated HepG2 cells in a fluidised-bed bioreactor. It may be noted that this BAL design could be adapted for any proliferating human liver cell-line available in the future. Our calculations - based as indicated on the requirement to provide 30-50% of liver mass - suggest that scaling up for human could provide the required 7×10^10^ to ∼1×10^11^ cells in 1600–2000 ml, a volume compatible with clinical use in a bioreactor.

In order to test the potential for clinical translation, experiments were organised with production of the BAL spheroids at a central facility and subsequently transportation to a distant hepatology centre, reflecting the way in which it would required to be used in practice. This is the way that clinical hepatology services are organised in many parts of the world. A robust and exacting system was established which required transport of more than 6000 miles by air and road to where the ALF model was set up by developing a short term cold chain enabling transport over 48 h at ambient temperature prior to use. Whilst this will not provide long-term storage at cryo-temperatures, it would be sufficient for delivery to any hospital that requires it; the perfluorodecalin required is available in GMP clinical grade, and is sterilisable by autoclaving [42]. For the future, we have demonstrated previously that the functional unit of this biomass, the alginate bead containing HepG2 cell-spheroids, can be cryopreserved with recovery of function within 48 hours, a timeframe suitable for subsequent clinical use [43], [44]. To our knowledge no other bioreactor design enables the biomass to be cryopreserved at optimal function prior to use, enabling an “off the shelf” product to treat patients. Moreover, we have recently utilised a non-liquid nitrogen cryocooler to effect the cryopreservation with encouraging results [45], further confirming this bioreactor design to be compatible with clinical use in a worldwide setting.

Further work to enable a disposable cryopreservable chamber for clinical scale biomass cryopreservation is ongoing. We are now in the process of refining design and manufacture of this system to GMP to provide a clinically ready bioartificial liver machine.

## Supporting Information

Figure S1 **Haemodynamic data in pigs with ischaemic acute liver failure treat with control or cell-bead BAL.** Haemodynamic data was obtained using a PiccoPlus monitor and picco software for data collection. Solid lines are animals in Group 1 (cell-bead treated); dashed lines are animal treated with empty bead non-functional control BAL. **A&B** show Mean Arterial Pressure in mmHg; **C&D** show cardiac output represented by PCCI in L/Min/m^3^; **E&F** show Systemic Vascular Resistance (SVRI) in dyns/second/cm^2^. Each is shown in time (hours) after ischaemia insult during BAL treatment.
(TIF)

Figure S2 **Further haemodynamic variables.** **2A**) Extravascular lung water was measured using the Picco plus machine at intervals after BAL addition. The average values were lower in the cell-bead treated group compared with control group indicating less fluid overload in the treated group. Extravascular lung water, a measure of oedema, is important during treatment of liver failure. **2B**) cerebral perfusion pressure did not differ between control and treated groups.
(TIF)

Figure S3 **Histology of liver after ischaemic damage.** **A**) liver x4-scale-bar-500 µm, **B**) Liver x40, **C&D**) brain x40; H&E. scale-bar 50 um.
(TIF)
