# Supplementary figures and images for: Evaluation of Encapsulated Liver Cell Spheroids in a Fluidised-Bed Bioartificial Liver for Treatment of Ischaemic Acute Liver Failure in Pigs in a Translational Setting
Source: PLoS One. 2013 Dec 18;8(12):e82312. doi: 10.1371/journal.pone.0082312 (PMC3867376; doi:10.1371/journal.pone.0082312)

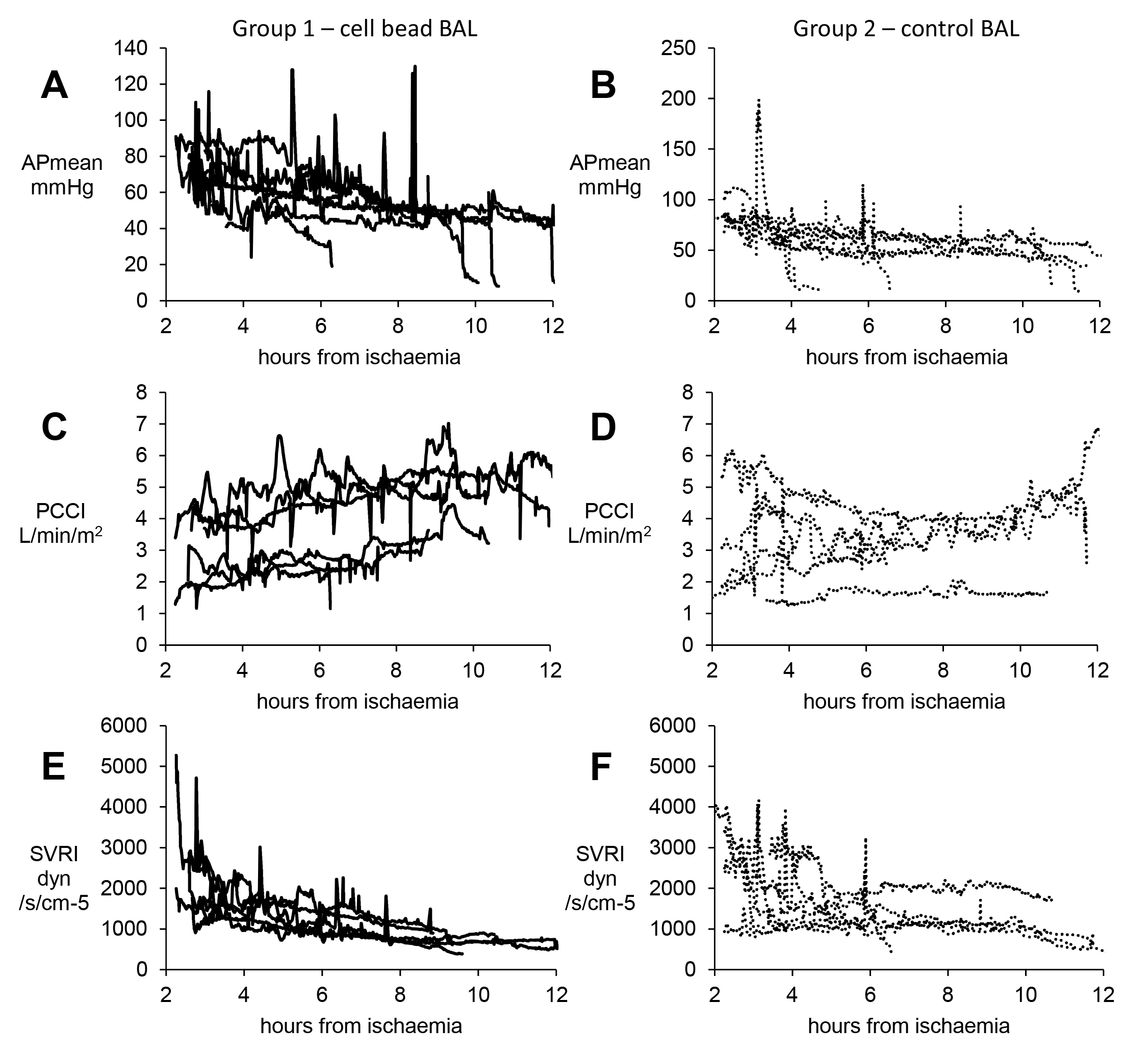

Supplement: Figure S1 — Haemodynamic data in pigs with ischaemic acute liver failure treat with control or cell-bead BAL. Haemodynamic data was obtained using a PiccoPlus monitor and picco software for data collection. Solid lines are animals in Group 1 (cell-bead treated); dashed lines are animal treated with empty bead non-functional control BAL. A&B show Mean Arterial Pressure in mmHg; C&D show cardiac output represented by PCCI in L/Min/m3; E&F show Systemic Vascular Resistance (SVRI) in dyns/second/cm2. Each is shown in time (hours) after ischaemia insult during BAL treatment. (TIF) [file pone.0082312.s001.tif]

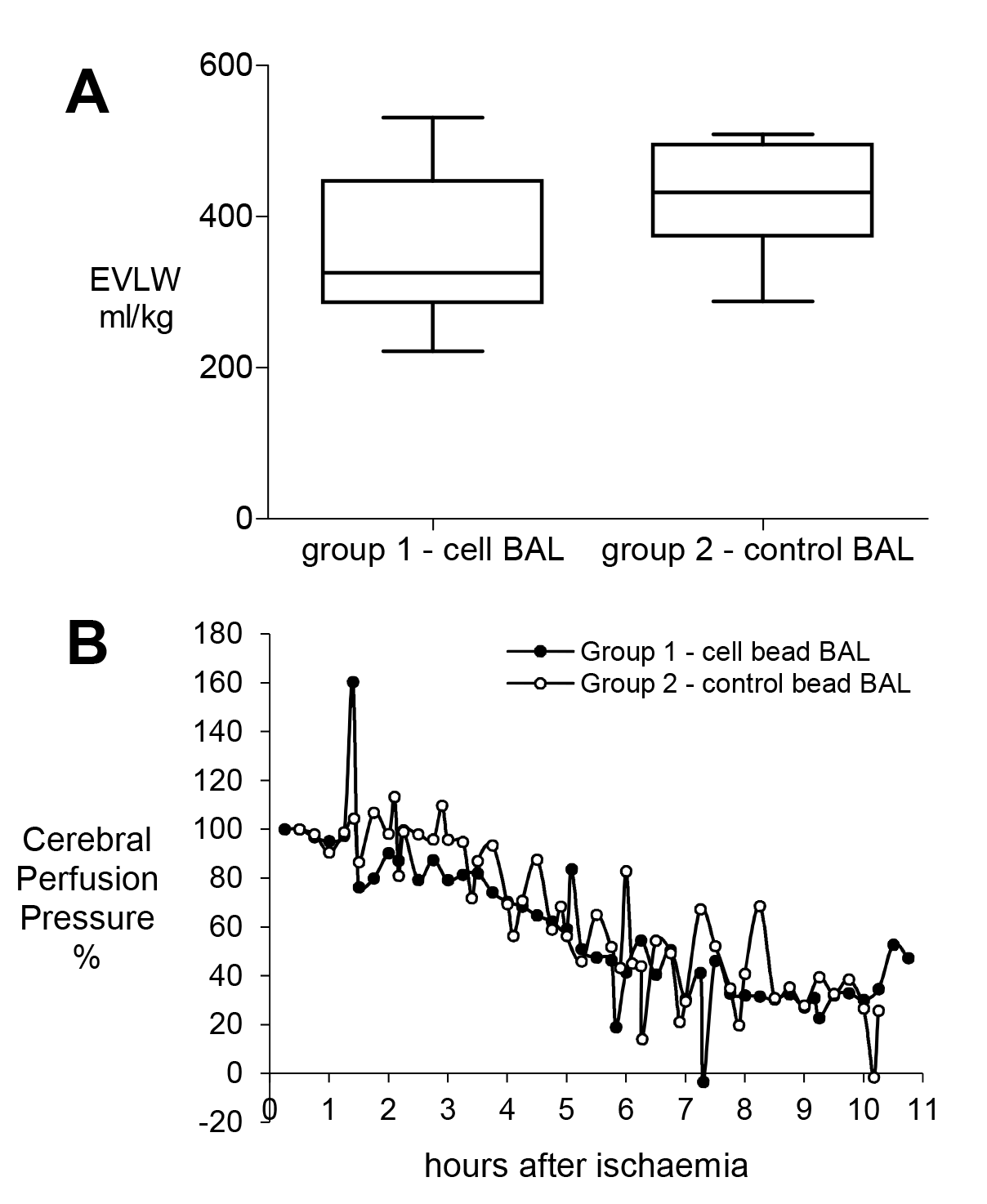

Supplement: Figure S2 — Further haemodynamic variables. 2A) Extravascular lung water was measured using the Picco plus machine at intervals after BAL addition. The average values were lower in the cell-bead treated group compared with control group indicating less fluid overload in the treated group. Extravascular lung water, a measure of oedema, is important during treatment of liver failure. 2B) cerebral perfusion pressure did not differ between control and treated groups. (TIF) [file pone.0082312.s002.tif]

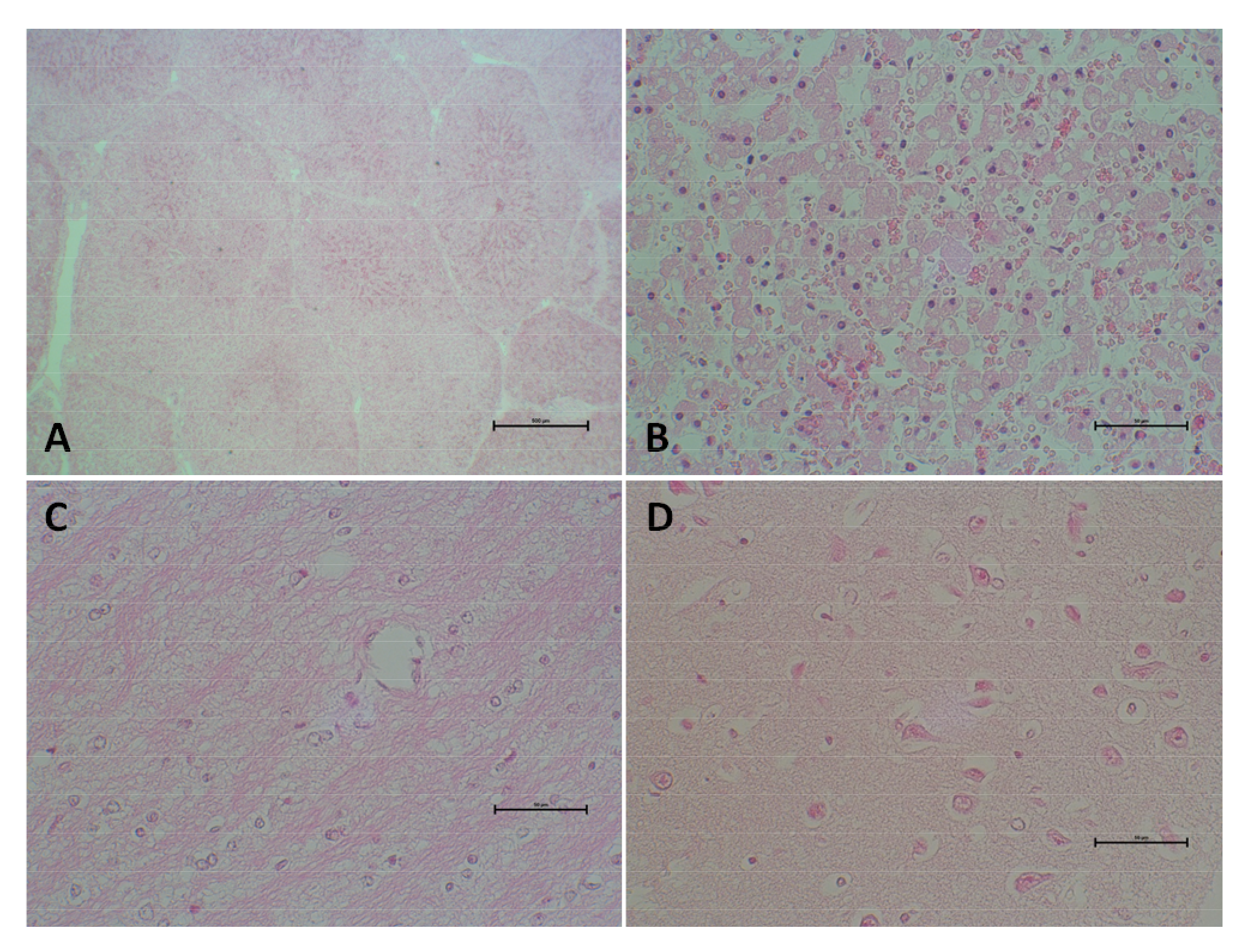

Supplement: Figure S3 — Histology of liver after ischaemic damage. A) liver x4-scale-bar-500 µm, B) Liver x40, C&D) brain x40; H&E. scale-bar 50 um. (TIF) [file pone.0082312.s003.tif]
